# Latest Advances in Preservation Technology for Fresh Fruit and Vegetables

**DOI:** 10.3390/foods11203236

**Published:** 2022-10-17

**Authors:** Peng Jin

**Affiliations:** College of Food Science and Technology, Nanjing Agricultural University, Nanjing 210095, China; pjin@njau.edu.cn

Fruit and vegetables contain abundant nutrients, as well as dietary and health benefits, and economic value, but suffer from shorter shelf life, declining quality, and rapid deterioration after harvest. Several preservation technologies can be used with fresh produce, such as chemical treatments, physical applications, and other approaches. This Special Issue aimed to collect the latest trends in diverse mechanisms of fresh product preservation and covered six chemical treatments: 1-Methylcyclopropene (1-MCP) [1], calcium chloride (CaCl_2_) [2], melatonin [3], hydrogen sulfide (H_2_S) [4], essential oils [5], lactic acid and acetic acid [6], in a broader postharvest quality attribute. Other treatments, such as cold shock, modified atmospheric packaging [7], and chlorophyllin-based photodynamic inactivation [8], were utilized in postharvest conditions.

1-MCP plays a crucial role in postharvest products, suppressing respiration and limiting ethylene peak. The study reported by Dai et al. showed that 100 μL L^−1^ concentration of 1-MCP treatment effectively induced antioxidant capacity in Chives (*Allium schoenosprasum* L.). The application of 1-MCP in this medicinal leafy vegetable considerably improved organosulfur profiles such as isoalliin and total-S-alk(en)ylcysteine sulfoxides (ACSOs) content [1]. The positive correlation between ACSOs and antioxidant enzyme activity is crucial in preserving the quality. Thus, it demonstrates the potential function of 1-MCP in maintaining quality while being stored [1]. In other conventional chemical treatments, Hou et al. studied the possible mechanism of CaCl_2_ in loquat (*Eriobotrya japonica*) fruit postharvest storage maintenance [2]. Several shreds of evidence show that Ca^2+^ is an essential trigger in the stress tolerance physiology of plants. The study showed that CaCl_2_ mitigated chilling injury and improved quality by modulating reactive oxygen species (ROS) homeostasis via higher antioxidant activities and scavenging capacity. Similarly, CaCl_2_ improves the ascorbate-glutathione (AsA-GSH) cycle and a higher expression level of corresponding genes, namely ascorbate peroxidase (*EjAPX*), glutathione reductase (*EjGR*), monodehydroascorbate reductase (*EjMDHR*), and dehydroascorbate reductase (*EjDHAR*) [2]. This explains the cold tolerance induced by CaCl_2_ treatment in loquat fruit.

As an organic preservative, melatonin is a vital stress regulator in plants physiology, with a possible role in the postharvest life of fruit and vegetables. The application of melatonin (600 μmol L^−1^) remarkably improved quality in postharvest guava (*Psidium guajava* L.) fruit [3]. The mechanisms involve enhancing enzymatic and non-enzymatic antioxidants, paralleled with regulating lipid metabolisms and defense-related enzymes such as chitinase, phenylalanine ammonia lyse, and 4-coumaric acid-CoA-ligase. It further helps maintain cellular integrity and boosts disease resistance, which results in the improvement of storage quality in guava [3]. In another gaseous preservative, H_2_S is utilized in horticultural products with low concentrations. The study proves that H_2_S effectively improved antioxidant activity and increased the activities of enzymes involved in energy metabolisms, including cytochrome C oxidase (CCO), succinate dehydrogenase (SDH), H^+^-ATPase, and Ca^2+^-ATPase [4]. Furthermore, H_2_S regulated proline metabolisms by enhancing key synthesizing enzymes, ornithine aminotransferase (OAT) and Δ^1^-pyrroline-5-carboxylate syntheses (P5CS) activities. Therefore, H_2_S could potentially alleviate chilling injury and maintain the quality of cucumber fruits [4]. In another study, an essential oil P-Anisaldehyde (PAA) was used for postharvest fruit preservation due to its antimicrobial properties [5]. The treatment of PAA improved the firmness, total soluble solids, total phenols, and flavonoids in postharvest pitaya fruit (*Hylocereus undatus*). In addition, hydrogen peroxide and superoxide anion production were limited by the essential oil treatment. Thus, the maintenance of fruit quality was attributed to the activated antioxidant enzymes namely superoxide dismutase (SOD), peroxidase (POD), catalase (CAT), and higher expressions of several corresponding genes encoding antioxidants enzymes (*HpSOD*, *HpPOD*, *HpCAT*, *HpAPX*, *HpGR*, *HpDHAR*, and *HpMDHAR*) [5]. In the proteomic study, 0.8% lactic acid + 0.2% acetic acid treatment was used to inhibit inhibit *Escherichia coli* O157:H7 contamination in fresh products [6]. This treatment managed to reduce membrane damage via a positive alteration of the stress response, catabolism, and molecular activities. The additional investigation suggested that the combined treatment of lactic acid and acetic acid could be used to preserve *E.coli*-susceptible fruit and vegetables [6].

Food waste postharvest poses a significant bottleneck, and an innovative reused approach is crucial in these areas. The antimicrobial properties of the 12 discarded vegetables were studied for distractive soft rot disease [9]. In tested vegetables, tomatoes showed a higher growth of bacteriocin—*Lactobacillus paracasei* WX322, which was induced by 10 days of fermentation of tomato juice. This bacteriocin is an antibacterial (precursor) polypeptide. The *Lactobacillus paracasei* WX322 causes severe damage to soft rot pathogen, namely *Pectobacterium cartovorum* (*Pcb* BZA12). Eventually, antimicrobial activity reduced soft rot and decay in cucumber, tomato, and green beans [9]. Therefore, innovative value-added organic preservatives could be applied to postharvest fruit and vegetables. In another disease-response-related report, the peptide PAF 56 (GHRKKWFW) shows a control mechanism in economically important citrus pathogenic fungi (*Penicillium digitatum*, *Penicillium italicum* and *Geotrichum citri-aurantii*) [10]. The pathways of action of the peptide on the spores of pathogen are primarily ascribed to the regulation of time-dependent cell membrane permeability and degree of membrane damage [10]. This provides insights for future research applications and commercial use.

In physical treatment approaches, the study showed that the combined effect of cold shock and passive atmosphere packaging (CS-PAP) significantly improved the quality and sensory attributes, as well as the nutritional value in postharvest cucumber fruit (*Cucumis sativus* L.) [7]. Thus, a related physical intervention could be applied for potential preservation under postharvest conditions [7]. Concerning another physical treatment, chlorophyllin-based photodynamic inactivation (ChI-PDI) affects the storage quality of pakchoi (*Brassica rapa subsp. chinensis*) [8]. The report demonstrated that chlorophyllin (1 × 10^–5^ mol L^−1^ and 405 nm light (22.27 J cm^−2^ per day)) treatment improved quality indicators, including vitamin C, total soluble solids and color preservation. Furthermore, it eliminated ROS accumulation by enhancing SOD and POD enzymes activities. The chlorophyllin treatment possibly attacked bacterial extracellular polysaccharides and extracellular proteins in vegetables. Hence, ChI-PDI is effectively used for fresh-cut preservation and displays good antimicrobial properties [8].

This editorial summarizes the selected latest chemical preservatives applications on fruits and vegetables for the regulation of antioxidant attributes, defense response, cold stress regulation, and subsequent shelf-life extension. Similarly, physical treatment can be applied to storage for preservation of fruits and to reduce distractive disease infestation.
